# From the Incretin Concept and the Discovery of GLP-1 to Today's Diabetes Therapy

**DOI:** 10.3389/fendo.2019.00260

**Published:** 2019-04-26

**Authors:** Jens Juul Holst

**Affiliations:** Department of Biomedical Sciences, Novo Nordisk Foundation Center for Basic Metabolic Research, University of Copenhagen, Copenhagen, Denmark

**Keywords:** proglucagon, glucagon, GIP, oxyntomodulin, glicentin

## Abstract

Researchers have been looking for insulin-stimulating factors for more than 100 years, and in the 1960ties it was definitively proven that the gastrointestinal tract releases important insulinotropic factors upon oral glucose intake, so-called incretin hormones. The first significant factor identified was the duodenal glucose-dependent insulinotropic polypeptide, GIP, which however, turned out not to stimulate insulin secretion in patients with type 2 diabetes. But resection experiments clearly indicated the presence of an additional incretin, and in 1986, an unexpected processing fragment of the recently identified glucagon precursor, proglucagon, namely truncated glucagon-like peptide 1 (GLP-1 7–36 amide), was isolated from the gut and found to both stimulate insulin secretion and inhibit glucagon secretion. The peptide also inhibited appetite and food intake. Unlike GIP, this peptide had preserved effects in patients with type 2 diabetes and it was soon documented to have powerful antidiabetic effects in clinical studies. Its utility was limited, however, because of an extremely short half-life in humans, but this problem had two solutions, both of which gave rise to important antidiabetic drugs: (1) orally active inhibitors of the enzyme dipeptidylpeptidase 4 (DPP-4 inhibitors), which was responsible for the rapid degradation; the inhibitors protect endogenous GLP-1 from degradation and thereby unfold its antidiabetic activity, and (2) long-acting injectable analogs of GLP-1 protected against DPP-4 degradation. Particularly, the latter, the GLP-1 receptor agonists, either alone or in various combinations, are so powerful that treatment allows more than 2/3 of type 2 diabetes patients to reach glycemic targets. In addition, these agents cause a weight loss which, with the most successful compounds, may exceed 10% of body weight. Most recently they have also been shown to be renoprotective and reduce cardiovascular risk and mortality.

Undoubtedly inspired by the discovery of the first hormone ever, secretin, in 1902 and gastrin in 1905, Moore et al. proposed in 1906 that the duodenal mucosa might secrete a hormonal substance, which would affect the disposal of glucose from the blood by acting as “a chemical excitant for the internal secretion of the pancreas” and tried to treat diabetes (not very successfully) by injections of gut extracts (1). But in 1929, Zunz and LaBarre (2) used cross-circulation experiments in dogs to demonstrate pancreas-dependent hypoglycemic effects after injection of upper intestinal extracts; such extracts clearly promoted pancreatic exocrine secretion (“excretin”), but the hypoglycemic effects via pancreatic endocrine secretion (insulin had been discovered in 1921) was thought to be due to an “incretin” (3). Another hypoglycemic duodenal extract, supposed to stimulate insulin secretion, was prepared by Heller in 1929 (4). However, it was not possible at the time to isolate and identify the “incretin” and it was only when it became possible to measure insulin (through the work of Yalow and Berson in the late 50ties) that the incretin effect could be further substantiated. With the insulin assay, it was possible to directly demonstrate increased insulin secretion after oral vs. intravenous glucose administration in spite of identical glucose concentrations, an effect which today is referred to as the “incretin effect” (5, 6). In elegant experiments, Perley and Kipnis in 1964 demonstrated that gastrointestinal, endocrine factors were responsible for the augmented insulin secretion (7) and also demonstrated that this mechanism was greatly impaired in “non-insulin requiring maturity onset diabetes.” The question then arose, which factors might be responsible, and the interest again turned toward the gastrointestinal hormones. Several hormones were tested for insulin-releasing activity and many of them were able to stimulate insulin secretion, so a set of criteria for assigning an incretin role to a hormone was established. First, the hormone had to be released after ingestion of glucose, since this stimulus defined the incretin effect. Secondly, the hormone should be capable of stimulating insulin secretion, when infused at rates resulting in plasma concentrations similar to those elicited by the oral glucose (8). This constitutes the “mimicry requirement.” Whether or not insulin secretion should be pre-stimulated with glucose was discussed, but given that the incretin definition included a comparison of insulin secretion during isoglycemic glucose challenges, it seems reasonable that the incretin candidate would stimulate insulin secretion primed by elevated plasma glucose concentrations. These criteria clearly excluded a number of hormones (e.g., cholecystokinin and gastrin), although it may still be argued that some of them could contribute to stimulation of insulin secretion under special circumstances (6). This applies, for instance, to secretin, which quite powerfully stimulates glucose-induced insulin secretion, but when infused in healthy volunteers reaching postprandial concentrations, it failed to do so (9). However, in situations with high concentrations of the hormone, which may be observed for instance after gastric bypass operations, plasma levels may be sufficient to stimulate insulin secretion. For cholecystokinin, there is an on-going debate as to which molecular component of it is the most prevalent and/or important and whether or not this component has sufficient insulinotropic effects in the very low concentrations at which it is circulating. Gastrin is the subject of a similar discussion (6). On the other hand, it may be argued the oral glucose is not a physiological stimulus, and that it would be more relevant to look at insulinotropic gastrointestinal factors released or activated after mixed meal ingestion. There is no doubt that neural elements may play a role for regulation of postprandial insulin secretion (10), but it can be clearly demonstrated that even in people with a denervated (transplanted) pancreas, there is an incretin effect (11). The endocrine component of the incretin effect, therefore, is relevant even if the neural component is disregarded. The absorbed nutrients may of course also influence insulin secretion themselves, and it is technically very difficult to control for the influence of all of the circulating nutrients and metabolites formed during meal ingestion. Interestingly, an incretin effect i.e., enhanced insulin response elicited during oral as opposed to intravenous intake, can be demonstrated not only for carbohydrate meals but also for protein and fat meals (12, 13). This observation is probably consistent with release of insulinotropic hormones during ingestion of all three macronutrients. All these stimulatory factors have been discussed under the common designation: the entero-insular axis (10). Clearly mapping of all components on the axis is complex, and an exhaustive mapping cannot be provided at present.

As already briefly alluded to, the incretin effect in its strict sense is quantified by performing an oral glucose tolerance test (typically with 75 g of glucose in healthy individuals; for people with diabetes lower amounts can be given, but this complicates the estimation because the magnitude of the effect depends on the amount of glucose ingested) (14, 15) on 1 day, with blood sampling for glucose and insulin and for investigative purposes hormone measurements. On a subsequent day, a glucose infusion is given, the rate of which is adjusted manually to copy the excursions from the oral day. This is not as difficult as it sounds (numerous descriptions in the literature). In addition to sampling for glucose determinations (every 5 min) blood is sampled at certain intervals for insulin and perhaps other hormone measurements. For accurate determination of insulin secretion, independent of varying hepatic insulin extraction, it may be useful to measure plasma levels of C-peptide, proinsulin's connecting peptide, which is not extracted in the human liver. The incretin effect may then be calculated as the difference between the integrated insulin responses to the oral and the intravenous glucose challenge and expressed in per cent of the response to the oral load (16). In healthy subjects, this usually amounts to up to 70%, which shows that the incretin effect is responsible for a major part of the postprandial insulin response. As mentioned, the effect, expressed in this way, is dependent on the amount of glucose ingested–with much lower values observed for lower amounts of glucose (14, 15).

An interesting, alternative way of looking at the gastrointestinal influence on the disposal of a glucose meal, is to calculate the amount of intravenously infused glucose required to mimick the oral challenge. For a 75 g oral glucose load this usually requires about 25 g of glucose. It follows that 2/3 of the ingested glucose is removed from the circulation by a mechanism that is dependent on the oral route. This estimate has been designated GIGD, gastro-intestinally mediated glucose disposal (17). Clearly, the predominant part of this is the stimulation of insulin secretion by gastrointestinal hormones, but suppressed glucagon secretion, for instance, could also play a role (which would further facilitate hepatic glucose uptake). Indeed, in patients with type 1 diabetes and no residual insulin secretion the GIGD may become negative, suggesting that for instance inhibition of glucagon secretion may be an important element of GIGD.

The ultimate question, however, is which hormones are in fact responsible for the incretin effect. Very soon after the discovery of *gastric inhibitory polypeptide* (GIP), by John Brown (18), he and John Dupre found that this new peptide powerfully enhances glucose-stimulated insulin secretion (19). Further research documented that GIP is released during oral glucose administration, and in careful mimicry experiments (20) it was established that GIP fulfills all requirements for acting as an incretin hormone. It was originally isolated on the basis of inhibitory effects on acid secretion in canine isolated gastric pouches, but this effect could not be reproduced under physiological circumstances in humans (21), and gradually the meaning of the acronym (GIP): Gastric Inhibitory Peptide, changed to *glucose-dependent insulinotropic polypeptide*. Animal experiments supported the essential function of GIP as an incretin hormone. Thus, immunoneutralization with antibodies against GIP significantly inhibited oral-glucose-stimulated insulin secretion (22). Later, after the introduction of the molecular biology methods and the cloning of the GIP receptor, mice with deletion of the GIP receptor did indeed exhibit reduced insulin responses (23), although the effect on these response and on oral glucose tolerance was small. However, by immunoneutralization in rats, it turned out to be impossible to completely remove the incretin effect (24), and experiments in which the incretin effect was compared with the GIP responses to glucose in people with various resections of the small intestine, confirmed that the incretin effect was not correlated with the GIP responses and upper small intestinal conservation, but rather with the preservation of distal small intestinal segments (25). These studies clearly pointed to the existence of another incretin hormone.

The interest now focussed on “gut glucagon” (26). Older studies had indicated that the gut (at least the canine gastric mucosa) produces a glucagon-like hyperglycemic substance (27), and antisera raised against the pancreatic alpha cell hormone glucagon, also identified endocrine cells scattered throughout the GI mucosa (28). However, accumulating immunochemical evidence and differences with respect to physico-chemical properties indicated that the “gut glucagon” was not identical to pancreatic glucagon, the structure of which had been deduced already in 1953 (29). Thus, antibodies directed against the C-terminus of glucagon would not react with these extracts unlike those directed against mid sequences of the peptide (30). Radioimmunoassays based on latter type of antibodies would also show secretion of substances with glucagon-like immunoreactivity (GLI) after oral glucose, whereas the C-terminal antisera would show decreasing concentrations, in agreement with Ohneda's demonstration of the glucose-dependency of pancreatic glucagon secretion (31). Eventually, the chemical nature of the gut GLI was revealed. There were two components, the smaller one being a fragment of the larger, but both containing the entire glucagon sequence (32, 33). The larger contained 69 amino acids of which residues 33–61 corresponded to the glucagon sequence(34), while the shorter form corresponded to residues 33–69 (33, 35). Thus, the smaller one, for which Dominique Bataille suggested the catching name oxyntomodulin, because of its alleged action on gastric oxyntic glands (36), was in effect a C-terminally extended form of glucagon. The larger one was designated glicentin, because Finn Sundby, who helped isolating the peptide, thought it contained 100 amino acids and combined “GLI” with “cent” + “in” to indicate its hormonal nature (37). Subsequently an N-terminal fragment corresponding to the first 30 amino acid of glicentin (Glicentin Related Pancreatic Polypeptide, GRPP) was isolated also from the pancreatic alpha cells, and was demonstrated to be released in parallel with glucagon (38), and glicentin was therefore proposed to represent at least a fragment of the hypothetical biosynthetic precursor of glucagon, proglucagon. Proglucagon would thus be processed in a tissue-specific, differential manner, giving rise to glucagon in the pancreas and to glicentin and oxyntomodulin in the so-called L-cells in the gut, the cells that harbored the glucagon immunoreactivity. Glicentin had no activity on the endocrine pancreas, but oxyntomodulin had a pronounced insulinotropic activity (39), and was therefore an incretin candidate. At the time, it was very difficult to measure oxyntomodulin specifically, because of the inherent cross-reaction that any antibody against oxyntomodulin would have with either glucagon or glicentin, but via chromatographic studies of extractable and circulating oxyntomodulin (40), combined with studies of its potency with respect to insulin secretion, it was eventually considered unlikely that this peptide was the putative second incretin hormone, a concept that still prevails (41).

On the other hand, it was also clear that glicentin did not represent the full proglucagon molecule. Evidence from cell free translation studies had indicated that proglucagon was considerably larger than glicentin (42), and using new molecular biology methods, Kay Lund, working with Joel Habener, managed to deduce the full structure of anglerfish proglucagon on the basis of material isolated from the compact islet organ, the Brockmann bodies of the anglerfish (43, 44). The proglucagon structure deduced from this work was longer indeed, and contained, apart from glucagon buried in a sequence with some resemblance to glicentin, an additional glucagon-like sequence [an account of the contributions from the Habener laboratory was published recently (45)]. On the basis of this, a race started to deduce the structure of mammalian proglucagon. In 1983, Graeme Bell published the amino acid sequence of a 160 amino acid protein, deduced from the mRNA sequence isolated from a hamster islet library (46), and later the same year, Bell and coworkers also published the full deduced structure of human proglucagon (47) (see Figure 1). On the basis of this, it was obvious that glicentin did indeed account for the entire N-terminus of proglucagon. However, the remaining part of the precursor contained not only one, but two glucagon-like structures framed by pairs of basic amino acid residues, the consensus cleavage sites for the recently characterized prohormone convertases (48) (Figure 1). The two glucagon-like sequences were rapidly synthesized by several groups, but neither the first, corresponding to the proglucagon 72–108 sequence or the second, corresponding to proglucagon 126–158 (it was predicted that the two final C-terminal amino acids, both basic, would be cleaved off during processing), had any effect on pancreatic insulin secretion (an early positive report of insulinotropic effects of PG 72–108 at very high concentrations (10^−7^ mol/L) (49) could not be reproduced by others). However, the new results made it possible to search for the actual products of proglucagon. Holst and coworkers in Copenhagen generated antibodies against peptides synthesized according to the deduced sequences, and used radioimmunoassays to analyse the occurrence, processing pattern, and secretion of immunoreactive glucagon-like peptides (GLPs) from the pancreas and the gut and found, in analogy with the differential processing of the N-terminal part of proglucagon (= glicentin), a similar differential processing also of the remaining part of proglucagon (50): in the pancreas, this resulted in formation and secretion of MPGF (45) (major proglucagon fragment), whereas in the gut there was both formation of and separate release of immunoreactive GLP-1 and GLP-2. Similar conclusions were reached by Svetlana Mojsov, working with Joel Habener in Boston (51). Because of the already mentioned lack of obvious biological activity of the two glucagon-like peptides, the group also used the immunoassays to search for immunoreactive peptides in extracts of the porcine and human gut using at the same time the perfused porcine pancreas as a bioassay for insulinotropic activity. This search resulted in identification of a highly insulinotropic peptide (52, 53). It turned out to be a truncated form of the predicted GLP-1, starting with residue no 78 and thus cleaved at a monobasic site. Furthermore, the C-terminal Gly from the predicted sequence was used as donor for amidation of the naturally occurring human peptide; the complete (human) structure of which therefore was proglucagon 78–107 amide (54). A synthetic replica of this peptide was indeed powerfully and potently insulinotropic (53). Observations by Svetlana Mojsov in Joel Habener's lab also supported a monobasic cleavage and a synthetic peptide corresponding to proglucagon 78–108 was indeed found to be insulinotropic (55). Natural GLP-2 was also isolated and sequenced in the Holst lab, and was found to correspond to PG 126–158 (56). Because of the original assumption that GLP-1 would be a peptide of 37 amino acids, the naturally occurring, biologically active form was often designated GLP-1 7–36 amide (or merely GLP-1), which is the preferred designation today. Importantly, small amounts of (inactive) proglucagon 72–107 amide or GLP-1 1–36 amide are produced in the pancreas (57), which has led to the erroneous assumption that active GLP-1 is also normally produced there. An account of these early developments was published recently (45).

**Figure 1:**
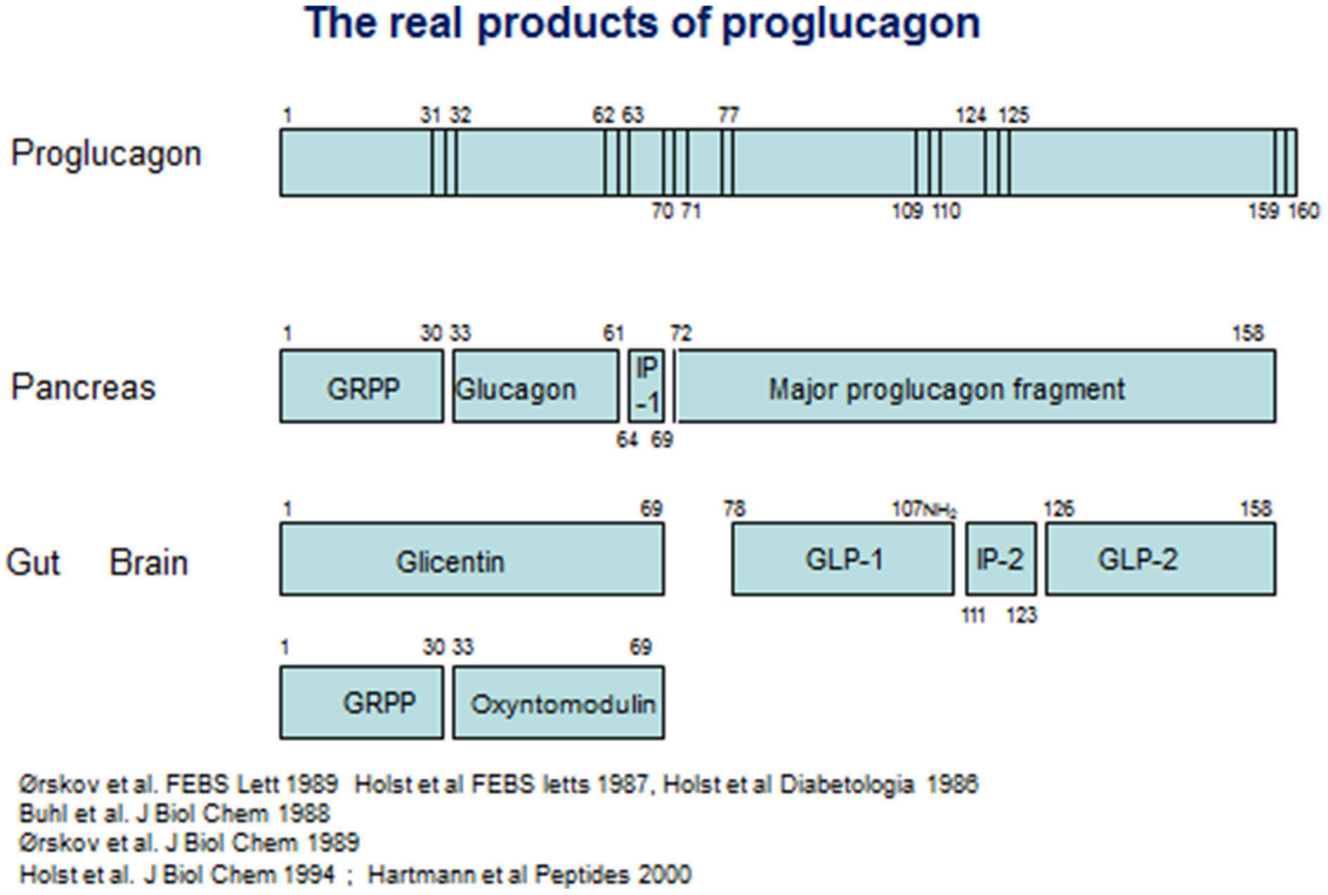
Products of proglucagon processing.

A first, the discovery of GLP-1 was met with moderate excitement; in fact, what was discovered, was yet another insulinotropic peptide from the gut (adding to the about 9 insulinotropic peptides already known to exist). In addition, incretin enthusiasts had suffered a serious disappointment, when it was observed, at about the same time, that GIP although being potently insulinotropic in healthy individuals was ineffective in patients with type 2 diabetes (58). This was interpreted to indicate that the beta cell failure of type 2 diabetes, i.e., the inability of the cells to respond to glucose with appropriately increased insulin secretion also would apply to the incretin hormones [in agreement with the demonstration by Perley and Kipnis (7) and Nauck et al. (59) of the loss of incretin effects in type 2 diabetes]. Nevertheless, with the help of the early radioimmunoassays, it was established that GLP-1 7–36 amide was indeed secreted from the gut in response to glucose ingestion (60) and in mimicry experiments its potential as an incretin was soon established (61). Also, renewed interest was aroused when it was discovered that GLP-1, unlike all the other insulinotropic peptides from the gut (including GIP but interestingly *not* including secretin) also was a potent inhibitor of glucagon secretion (62). So this new peptide from the gut had potential to influence blood glucose in two ways, both by stimulating glucose-induced insulin release and by inhibiting glucagon secretion, both of which would limit hepatic glucose production, the main driver of the fasting hyperglycemia of type 2 diabetes. Indeed, in subsequent studies with infusions of physiological amounts of GLP-1 both its insulinotropic and its glucagon-inhibitory effects (at fasting glucose concentrations!) as well as an ensuing reduction of hepatic glucose production were demonstrated in human volunteers (63); these experiments also showed that although glucose production was inhibited initially, plasma glucose concentrations only fell by 0.5–1.0 mmol/L in spite of prolonged infusion, because the insulinotropic effect of GLP-1 disappeared as glucose concentrations fell, demonstrating the glucose dependency of these actions. Indeed, in later clinical studies it was demonstrated that when it comes to the antidiabetic effects of GLP-1, the inhibition of glucagon secretion is at least as important as the stimulation of insulin secretion (64).

Inspired by the similarity of GLP-1 with glucagon and oxyntomodulin, it was relevant to look at other “glucagon-like” gastro-intestinal actions of GLP-1, and via extensive human studies it was soon established that GLP-1 was a physiological and powerful inhibitor of gastrointestinal secretion (both gastric and pancreatic) and motility (65), with a very strong inhibitory effect on gastric emptying (66). Most (all?) of these effects were apparently transmitted via inhibition of efferent vagus nerve activity (67, 68), providing early suggestions of powerful actions of GLP-1 via hindbrain and hypothalamic mechanisms. In agreement with the high density of L-cells in the distal part of the small intestine, from where the so-called ileal brake mechanism (upper gastrointestinal inhibition elicited by distal stimulation) is elicited, it appeared that GLP-1 might be one of the hormones behind it (69), sending inhibitory signals to the brain and proximal GI tract upon arrival of nutrients to the distal small intestine. To the extent that nutrients were also increased in the circulation at this time, GLP-1 would also by stimulation of insulin secretion promote the deposition of the nutrients while braking further intake. According to this view, the glucose-induced incretin function would mainly be exerted by the proximally located GIP, while GLP-1 would come into play after more excessive nutrient intake resulting in more distal exposure. Today, it has been possible to finally dissect the relative importance of the two hormones GIP and GLP-1 for the incretin effect in man; the tool making this progress possible was the development of specific and potent antagonists of the GLP-1 and GIP receptors (see below), that could be used in humans: exendin-9-39 for the GLP-1 receptor (70) and GIP 3–30 NH2 for the GIP receptor (71). In experiments with healthy volunteers, dual antagonism during oral glucose or mixed meal ingestion, revealed additive contributions of the two hormones on postprandial glucose excursions as expected, but regarding insulin secretion, removal of the GIP component resulted in the most pronounced reduction of insulin secretion, whereas glucagon secretion was only affected by the GLP-1 component.

In spite of the disappointment with GIP in T2DM, it was time to see whether the new incretin would have any effects in T2DM patients. The first study was done by Gutniak et al. (72) in Stockholm. They infused synthetic GLP-1 into both type 1 and type 2 diabetic patients and used the Biostator, an early artificial pancreas, to demonstrate that almost no insulin administration was needed to normalize blood glucose levels, when GLP-1 was infused. Almost simultaneously, Nauck et al demonstrated that infusions of GLP-1 during slightly hyperglycemic clamp conditions in both patients with type 2 diabetes and matched controls elicited almost similar insulin responses (whereas GIP infusions were ineffective) (73). In continued studies, intravenous infusions of GLP-1 in slightly supraphysiological amounts, were able to completely normalize fasting plasma glucose concentrations in patients with long standing T2DM admitted to hospital because of poor glycemic control (fasting glucose around 13 mmol/L) (74). The normalization was accompanied by increases in insulin and lowering of glucagon plasma concentrations, both of which returned to the original levels as glucose concentrations were getting normalized, illustrating the glucose dependency and safety (with respect to hypoglycaemia) of the GLP-1 infusion. These experiments marked the beginning of the era of the GLP-1 based therapies.

The preserved insulinotropic effect of GLP-1 in patients with T2DM may seem incompatible with the almost complete loss of incretin effect in these patients (75). Subsequent studies have shown that low physiological concentrations of GLP-1 are indeed ineffective in patients with T2DM (76), whereas larger slightly supraphysiological doses [like those employed by Nauck et al. (74)] could in fact normalize beta cell sensitivity to glucose (77); in contrast, GIP remains inactive in T2DM regardless of dose (77). The molecular explanation for this difference is still not known.

In the late 80ties, researchers began looking for the new glucagon-like peptides in the brain, inspired by the reports of immunoreactive glucagon in certain neurons of the brain (78), particularly in the nucleus of the solitary tract. These neurons also were immunoreactive for GLP-1 (79). In addition, binding studies suggested the presence of a large number of putative receptors for GLP-1 not only in the beta cells of the pancreatic islets but also in the brain (80). In 1992, Bernard Thorens managed to clone the single GLP-1 receptor (81), making it possible to move from GLP-1 binding to receptor expression which confirmed the expression pattern in the brain (82, 83). A likely interpretation of these findings was that the brain receptors were targets of projections from the GLP-1 neurons in the nucleus of the solitary tract in the brain stem (84). In addition, inhibition of food intake could be elicited by intracerebroventricular (ICV) administration of GLP-1 to rats (85, 86). But were these observations relevant for peripheral GLP-1? Would peripheral GLP-1 be able to reach nuclei in the brain and what might be the effect of this? It was soon demonstrated that leaks in the blood barrier notably in the area postrema, the subfornical organ and the median eminence would not only allow entry of GLP-1 into these regions of the brain (87), but there was also dense receptor expression at these sites suggesting peripheral GLP-1 might also have actions on the brain (80), and in 1998 it was demonstrated that peripheral intravenous infusions of physiological amounts of GLP-1 would lead to decreased appetite and inhibition of food intake (88). It remains unclear whether there is a relationship between the central actions of peripheral GLP-1 and the GLP-1-producing neurons in the brain stem, but it is clear that one of the most important actions of peripheral GLP-1 is to regulate food intake in agreement with its “ileal brake” function.

The demonstration of the GLP-1 receptor and its expression on the beta cells was of course consistent with its powerful insulinotropic effects (89) and was soon followed by a large number of cell biological studies of its effect on beta cell biology (90). Thus, the receptor was predominantly Gs coupled leading to cAMP production and activation of protein kinase A, and, as shown later, activation of epac2, the guanine nucleotide exchange protein (91). In various models, GLP-1 was also able to stimulate beta cell replication [although this was dependent on the age of the islet cells (92)] as well as neogenesis (from ductal precursors) (93) and importantly GLP-1 also inhibited apoptosis of human beta cells, induced for instance by fatty acids or cytokines (94). Together, these observations indicated that GLP-1 may have protective effects on beta cells, and raised hopes for long lasting and perhaps disease modifying effects of GLP-1 in diabetes therapy. Today, long lasting trials have shown that the treatment effect is indeed durable (95), but whether or not this can be interpreted as a beta cell protective effect remains uncertain.

Nevertheless, treatment of diabetes was obviously of great interest, but how to do it? Effects of single s.c. GLP-1 injections were extremely short lasting (the plasma half-life of the peptide in humans after iv. injection is around 2 min) (96). Oral administration was out of the question [and effects of buccal administration equally short-lasting (97)]. Therefore, in order to investigate whether long term treatment with GLP-1 was feasible at all, GLP-1 was given as a continuous subcutaneous infusion for 6 weeks in a trial in obese people with severe Type 2 diabetes (98). Fortunately, no tachyphylaxis was observed: GLP-1 therapy reduced fasting and mean plasma glucose by 4.3 and 5.5 mmol/L; glycated hemoglobin by 1.3%; and body weight by 2 kg. Moreover, insulin sensitivity and beta cell function, assessed by clamp studies, were greatly improved. Importantly, no significant side effects were recorded, providing proof of concept for GLP-1 therapy in subjects with T2D. It was now clear that GLP-1 based therapies had tremendous potential.

Of course, continuous subcutaneous infusion was not an attractive approach for practical clinical application, so something had to be done. The almost immediate degradation of GLP-1 in the body raised the question of the mechanisms involved, and inspired by early studies by the German enzymologist, Mentlein (99), Deacon and Holst identified the enzyme dipeptidyl peptidase 4 as the enzyme responsible for the degradation of GLP-1 in plasma (100) and demonstrated that this could be prevented with inhibitors of the enzyme; in analogy with the use of ACE inhibitors for hypertension, and based on clinical studies they proposed the use of DPP-4 inhibitors for the therapy of T2DM (101). In model experiments in pigs, they demonstrated that it was possible to completely protect infused GLP-1 against DPP-4 mediated degradation, and that this protection resulted in marked increase in the insulin response to glucose and GLP-1 (102). These and other studies gave impetus to the clinical development of the inhibitors of DPP-4 for diabetes therapy, and in a pivotal proof-of concept study by Ahren and coworkers in 2004, vildagliptin, developed by researchers at Novartis, showed hemoglobin A1c improvements to target levels of 7% in a 1-year trial, whereas a significant rise was observed in the placebo group (103). The clinical aspects of the DPP-4 inhibitors, which are now used world-wide for diabetes treatment, will be discussed by Deacon, Ahren and others in other contributors to this Research Topic.

Because the cleavage activity of DPP-4 is depending on the presence of a penultimate Pro or Ala at the N-terminus, analogs of GLP-1 with substitutions at this position should be protected, and analogs with Ser, Val, or alpha-aminobutyric acid remained biologically active (104), so this problem could easily be solved, but the substitutions only increased the half-life from 1.5 to 4–5 min (104), the reason being a rapid extraction of GLP-1 in the kidneys (105), and probably also other enzymatic attacks (106). Clearly, additional modifications were required to produce a clinically effective analog of GLP-1. This is where exendin-4 entered the stage. Exendin-4 is a peptide isolated from the saliva (not the venom) of the Gila Monster (Heloderma Suspectum) (107). It consists of 39 amino acids with the 30 N-terminal residues showing 53% homology with mammalian GLP-1. Exendin-4 is not the GLP-1 of the Gila Monster [it has its own GLP-1 with a much higher homology, in agreement with the finding that the sequence is highly conserved among species with 100% identity among all mammals so far investigated (108)], but is nevertheless a full and potent agonist of the mammalian GLP-1 receptor. The fascinating story of how this reptile peptide made it to the diabetes market has been described previously (109). Exendin-4 is not sensitive to DPP-4 activity, and is not extracted in the kidneys, but freely filtered resulting in an i.v. half-life of around 30 min. Upon subcutaneous injection there is an adequate exposure of this peptide for up to 5 h (110), which suffices for meal-related clinical activity. Since the peptide, in analogy with GLP-1, powerfully inhibits gastric empting, the result is absolutely no rise in plasma glucose after meal ingestion (111). On this background, a synthetic replica of exendin-4, exenatide, was developed for clinical T2DM therapy with two daily injections (110). Clinical trials were satisfactory and the peptide was approved and marketed for therapy as the first GLP-1 receptor agonist in 2005. Another, slightly modified synthetic version of exendin-4, lixisenatide (112), was subsequently launched and designated for once daily use, but in fact the actions and pharmacokinetics of the two peptides are identical. The second GLP-1 agonist to reach the market [2009–10] was liraglutide (113), consisting of mammalian GLP-1 to which was attached a palmitic acid chain via a linker coupled to the Lys residue 26. As expected from the experience with a similarly acylated peptide, levemir, this resulted in albumin binding, and near elimination of renal filtration as well as relative DPP-4 resistance. The half-life after s.c. injection was now around 12 h, suitable for once daily injection, but with a high average exposure during chronic treatment (114). The effectiveness, particularly with respect to fasting glucose concentrations of the long-acting analog was, as expected, considerably improved. The biological activity, also on other targets (115), was not diminished, and liraglutide has also been approved for obesity treatment (116).

By covalent (albiglutide, dulaglutide) or no-covalent (semaglutide) coupling to larger molecules with slow clearance, analogs with even more prolonged activity have been produced so that there are now several once-weekly agonists on the market. Also exenatide is available in a once weekly version, in which the molecule is slowly liberated from a subcutaneously injected depot. The most radical solution was adopted by the company Intarcia, whose product Itca 650 comprises exenatide administered in an osmotic minipump the size of a match, and similar to those used for animals, but capable of providing a constant delivery of exenatide for 6–12 months after subcutaneous implantation (117).

In addition to the actions of GLP-1 discussed above, the long acting analogs have also in long-term cardiovascular outcome studies been demonstrated to result in reduced cardiovascular risk, so far only in patients with both T2DM and cardiovascular risk (95, 118, 119), but emerging evidence suggests that this may also apply to individuals without established heart disease. The mechanism involved is not known, but the cardiovascular actions will importantly support the future use of GLP-1 receptor agonists, which are already generally recommended as second line therapies for patients with metformin failure (120).

The GLP-1 RAs are now generally recommended for second line therapy after metformin in many patients with T2DM (120), but the truth is that rather few patients actually use them. A number of explanations for this may be offered: (1) they are injectables which may represent a problem for some; (2) They have a bad reputation after accusations that they would cause pancreatitis, pancreatic and thyroid cancers and C-cell hyperplasia although these were unfounded and were not confirmed in the large CVOTs; (3) the gastrointestinal side effects (nausea, vomiting, bile system complications) although not frequent with newer up-titration protocols; (4) the fact that the weight effect is generally small and that the effect flattens out after some time, which is experienced as disappointing; (5) the fact that treatment interferes with appetite and the pleasure of eating, perhaps due to interference by the GLP-1 RAs with the reward system of the CNS (121), which may be experienced negatively by some; but finally (6) the price, which is dramatically higher than that of metformin and the sulfonylureas. The latter issue has made the WHO not to recommend the use of GLP-1RAs in their most recent set of recommendations (122). The first GLP-1RAs will go off patent in the early 2020-ties and this will undoubtedly lower the price. It is also possible that the likely oral availability of a GLP-1RA (123) will promote the future use. The DPP-4 inhibitors which have already conquered a massive share of the global market are likely to remain keep their status in the years to come in spite of their lesser efficacy for two important reasons (1) their price is gradually falling and (2) their unusually benign side effect profile (124).

## Author Contributions

The author confirms being the sole contributor of this work and has approved it for publication.

### Conflict of Interest Statement

The author declares that the research was conducted in the absence of any commercial or financial relationships that could be construed as a potential conflict of interest.
